# Temporal trends, diagnostics, and outcomes of pediatric nontuberculous mycobacterial disease in Slovakia (2017–2024)

**DOI:** 10.3389/fpubh.2026.1713591

**Published:** 2026-04-09

**Authors:** Matúš Dohál, Peter Kunč, Jaroslav Fábry, Igor Porvazník, Ivan Solovič, Michaela Krivošová, Juraj Mokrý

**Affiliations:** 1 Biomedical Centre Martin, Jessenius Faculty of Medicine in Martin, Comenius University, Bratislava, Slovakia; 2 Clinic of Pediatric Respiratory Diseases and Tuberculosis, National Institute of Pediatric Tuberculosis and Respiratory Diseases, Dolný Smokovec, Jessenius Faculty of Medicine in Martin, Comenius University, Bratislava, Slovakia; 3 Department of Pathological Physiology, Jessenius Faculty of Medicine in Martin, Comenius University, Bratislava, Slovakia; 4 National Institute of Tuberculosis Lung Diseases and Thoracic Surgery, Vyšné Hágy, Slovakia; 5 Faculty of Health, Catholic University, Ružomberok, Slovakia; 6 Department of Pharmacology, Jessenius Faculty of Medicine in Martin, Comenius University, Bratislava, Slovakia

**Keywords:** diagnostics, disease, epidemiology, *M. avium*, nontuberculous mycobacteria, pediatrics, treatment

## Abstract

**Background:**

Nontuberculous mycobacteria (NTM) are rare but emerging pathogens in pediatric populations, particularly in countries where BCG vaccination has been discontinued. Their diagnosis is often delayed due to nonspecific symptoms and limited microbiological sensitivity.

**Methods:**

We conducted a retrospective cohort study of all pediatric patients with confirmed or clinically probable NTM disease, diagnosed at the National Institute for Pediatric Tuberculosis and Respiratory Diseases in Slovakia between 2017 and 2024. Medical records from 2012 to 2016 were screened, but no cases fulfilling inclusion criteria were identified. Clinical characteristics, diagnostic approaches, therapeutic strategies, and patient outcomes were systematically evaluated.

**Results:**

In total, 30 patients were diagnosed. The majority of cases (27/30) involved cervical lymphadenitis (including two with concurrent pulmonary involvement), most commonly affecting children between 1 and 2 years. In addition, one patient was diagnosed with a pulmonary form of NTM disease, one with NTM-associated osteomyelitis, and one with a skin and soft tissue infection. *Mycobacterium avium* complex was the most frequently identified species, detected in 8 patients. Bacteriological confirmation was achieved in 53.3% of cases, while histological evidence of granulomatous inflammation was found in 86.7%. The median time to diagnosis was 45 days (IQR: 31–109.5), with longer delays in culture-negative patients (*p* = 0.0648). Surgical excision was performed in 25 of 27 patients with lymphadenitis, 60% received adjunctive antibiotic therapy. Follow-up data were available for 19 patients: 15 had full recovery, 3 experienced recurrent upper respiratory tract infections, and 1 immunocompromised patient died from miliary tuberculosis.

**Conclusion:**

This is the first national study on pediatric NTM disease in a post-BCG vaccination era in Slovakia. Despite centralization of care, diagnostic delays were common, particularly in bacteriologically negative cases. These findings underscore the need for early tissue sampling, comprehensive microbiological evaluation, and interdisciplinary collaboration to improve diagnostic efficiency.

## 1 Introduction

Nontuberculous mycobacterial (NTM) diseases represent an increasingly recognized clinical challenge in pediatric medicine. The prevalence of NTM disease in pediatric populations demonstrates significant geographic variation, with reported incidence rates ranging from 0.6 to 5.36 cases per 100,000 children annually across different countries (1, 2). NTM are ubiquitous environmental bacteria found in water, dust, soil and various ecological nitches (3). Children’s behaviours, particularly those in 1–5-year age group, increase exposure risk through activities such as playing in sandboxes, placing contaminated objects in their mouths or direct contact with natural water sources or swimming pools (4–6). This is further supported by the fact that the highest incidence of cervicofacial lymphadenitis (the most common form of NTM disease), occurs in this age group (7, 8). The increase in incidence has also been documented in older children and adolescents, often in association with underlying conditions such as cystic fibrosis (9, 10).

NTM diseases in children present with four distinct clinical syndromes, each with characteristic features and epidemiologic patterns. The predominant manifestation varies by age, immune status, and underlying conditions. Cervicofacial lymphadenitis (the most commonly caused by *M. avium* complex) presenting as unilateral, painless, progressive lymph node enlargement in the neck region is the most common clinical manifestation of NTM disease in immunocompetent children, accounting for more than 70% of all pediatric NTM diseases (2, 7). Importantly, systemic symptoms such as fever, weight loss, or malaise are typically absent, distinguishing NTM lymphadenitis from tuberculosis (11). Pulmonary NTM disease in children represents a more complex clinical entity, often occurring in association with underlying lung conditions. In cystic fibrosis patients, NTM prevalence has shown increasing trends (9). The most frequently isolated species include *M. abscessus* and *M. avium* complex (12, 13). Less common manifestations of NTM disease in children include skin and soft tissue infections (SSTIs), often arising after trauma or cosmetic interventions, and disseminated disease, which predominantly occurs in severely immunocompromised individuals, such as those with advanced HIV infection or underlying primary immunodeficiencies (14). The prognosis for disseminated NTM disease is generally poor, with the mortality rate reaching 33% (15).

The diagnosis of NTM diseases in children requires a combination of clinical, radiological, and microbiological criteria. Clinical suspicion should be raised in children presenting with characteristic syndromes, particularly chronic lymphadenitis in young children or pulmonary symptoms in those underlying lung disease (16). However, the diagnostics is often delayed. The time from the onset of symptoms to diagnosis may range from several weeks to several months, particularly due to the indolent course of the infection and its nonspecific clinical presentation.

This study aimed to assess clinical manifestations, diagnostic delay and accuracy, treatment strategies, and outcomes in pediatric patients with NTM disease referred to the National Institute of Pediatric Tuberculosis and Respiratory Diseases in Slovakia over the period 2017–2024, following a preliminary screening of records from 2012 to 2016 in which no eligible cases were identified.

## 2 Materials and methods

### 2.1 Patients

A retrospective study was conducted using data extracted from electronic medical records of patients hospitalized at the National Institute for Pediatric Tuberculosis and Respiratory Diseases in Slovakia. Patients included in the study had been screened based on ICD codes for any form of mycobacteriosis during the period from 2012 to 2024. No patients from 2012 to 2016 fulfilled the diagnostic criteria for microbiologically confirmed or clinically probable NTM disease. Therefore, the analytical cohort includes only patients diagnosed between 2017 and 2024.

The following patient data were collected: gender, age, clinical phenotype (categorized into cervical lymphadenitis, cervical lymphadenitis with pulmonary involvement, pulmonary NTM disease, osteomyelitis, and SSTI), bacteriological confirmation, histological evidence (defined as the presence of granulomas with or without necrosis), TB-vaccination status, presence of underlying conditions (chronic disease or immunodeficiency), environmental exposure (defined as caregiver-reported contact with domestic or farm animals and/or exposure to untreated water sources during routine clinical evaluation), diagnostic delay (days between the first documented clinical symptom attributable to NTM disease and the initiation of targeted treatment), results of immunological tests for TB (QuantiFERON-TB Gold assay, QIAGEN, Hilden, Germany, and the Mantoux tuberculin skin test). Chronic disease was defined as any long-term comorbidity documented in the medical record (e.g., asthma, cystic fibrosis, auto inflammatory or hematologic disorders). Immunodeficiency was evaluated as an independent category and included both primary immunodeficiency disorders and secondary or functional immune abnormalities identified by immunological testing or clinical assessment.

The data used in this article were fully anonymized in accordance with ethical guidelines and the General Data Protection Regulation (GDPR) to ensure the privacy and confidentiality of all individuals involved.

### 2.2 Diagnostic criteria

For extrapulmonary NTM disease (e.g., lymphadenitis, SSTI, disseminated disease), patients were classified into two diagnostic categories: microbiologically confirmed NTM disease and clinically probable NTM disease. Microbiological confirmation required detection of NTM by culture and/or PCR from a clinically relevant specimen, with exclusion of *Mycobacterium tuberculosis*. Clinically probable NTM disease was defined as a compatible clinical presentation (most commonly cervicofacial lymphadenitis), histological evidence of granulomatous inflammation, exclusion of *M. tuberculosis*, and multidisciplinary expert consensus at a national referral center, despite negative microbiological results.

A variety of clinical specimens were analyzed for the presence of NTM depending on the suspected site of infection. Lymph node specimens obtained by fine-needle aspiration or complete surgical excision were considered normally sterile material and were therefore not routinely subjected to chemical decontamination. Aspirated material was examined directly, whereas tissue samples were mechanically homogenized in sterile saline under standard biosafety conditions. Selective decontamination using N-acetyl-L-cysteine–sodium hydroxide (NALC–NaOH) was performed only in cases where contamination was suspected or when excessive background flora was observed on microscopic evaluation. In contrast, non-sterile respiratory and other clinical specimens, including bronchoalveolar lavage (BAL), gastric aspirates, laryngeal swabs, sputum, wound swabs, and bone marrow samples, were routinely decontaminated using the NALC–NaOH method prior to culture and molecular testing (in accordance with standard laboratory protocols), concentrated by centrifugation, neutralized, and washed with phosphate-buffered saline prior to further testing. Smear microscopy using Ziehl–Neelsen staining, culture on Löwenstein–Jensen medium, and molecular detection of mycobacteria by the Anyplex™ MTB/NTM assay (Seegene, Korea) were performed for all samples. In culture-positive samples, line-probe assays (GenoType Mycobacterium CM v2.0, AS v1.0, and NTM-DR v1.0; Hain Lifescience GmbH, Nehren, Germany) were used for definitive species or subspecies identification.

For pulmonary NTM disease, diagnosis was based on the clinical, radiological, and microbiological criteria recommended by the American Thoracic Society/Infectious Diseases Society of America (ATS/IDSA) (17).

### 2.3 Statistics

For categorical variables, absolute frequencies were calculated. For continuous variables, descriptive statistics included medians and interquartile ranges (IQRs). Diagnostic delay was defined as the time from the first documented symptom attributable to NTM disease to the initiation of the first NTM-directed intervention. Depending on the clinical phenotype, this intervention consisted of surgical excision or biopsy (for lymphadenitis-dominant disease) or initiation of antimycobacterial therapy. Normality of continuous variables was assessed using the Shapiro–Wilk test. As the distribution of diagnostic delays was non-normal and variances between groups were unequal, parametric tests were not appropriate. Therefore, comparisons between bacteriologically confirmed and non-confirmed cases were performed using the Mann–Whitney *U* test. A *p*-value ≤ 0.05 was considered statistically significant. For statistical analyses, GraphPad Prism 8.0.1 (GraphPad, San Diego, CA, USA) was utilized.

Microbiological positivity was defined at the patient level as the presence of at least one positive microbiological test (PCR, culture, or smear) from any clinical specimen. Individual patients could contribute multiple specimens; however, each patient was counted only once for patient-level analyses.

Specimen-level diagnostic yield was analyzed descriptively to compare the performance of different specimen types and laboratory methods.

## 3 Results

### 3.1 Patients

**Figure 1:**
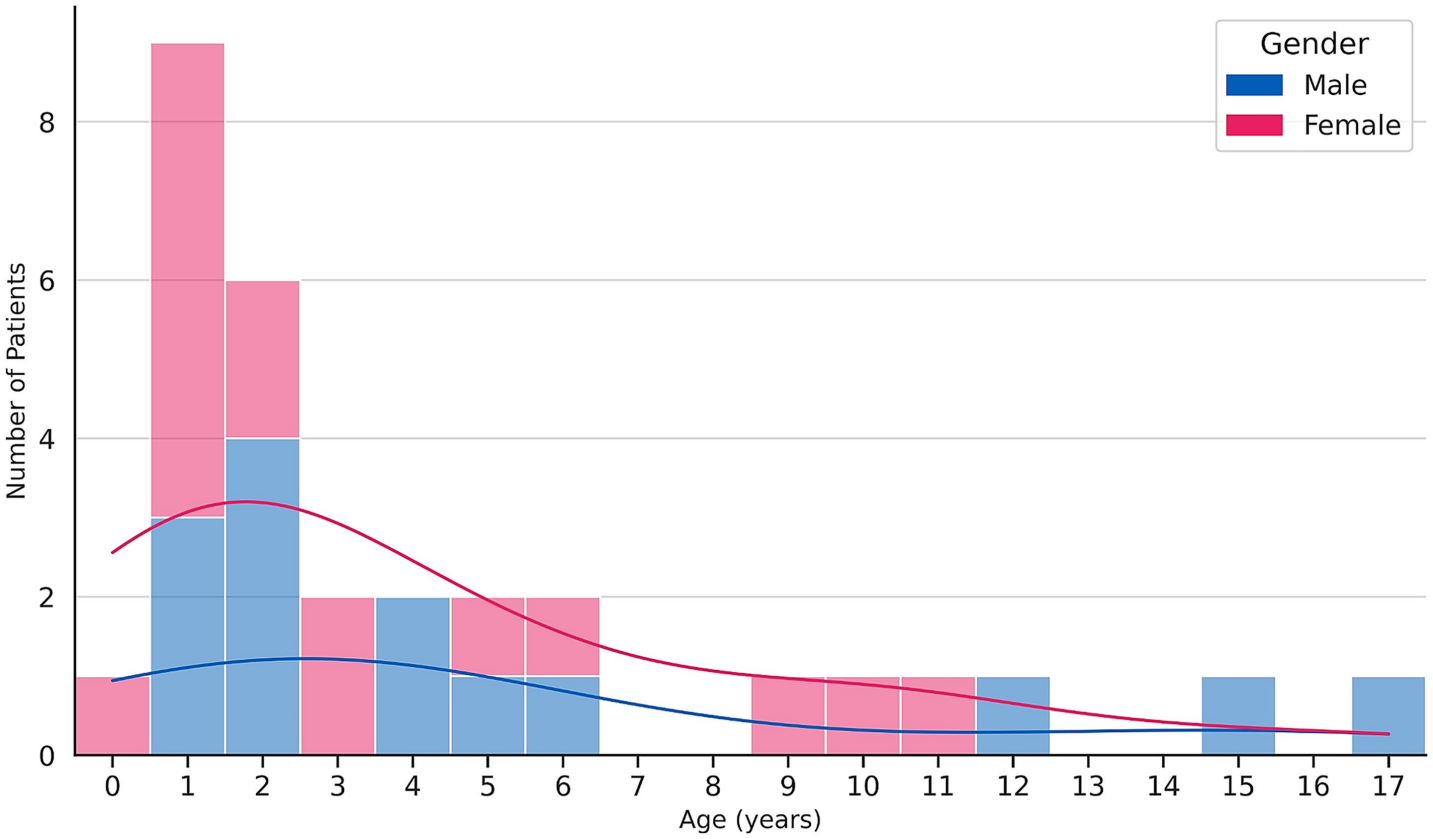
Age and gender distribution of patients diagnosed with NTM disease in Slovakia between 2017 and 2024. A kernel density estimate curve is overlaid to illustrate the smoothed age distribution.

A total of 30 pediatric patients with microbiologically confirmed (*n* = 16) or clinically probable (*n* = 14) NTM disease were identified during the years 2017–2024. No confirmed NTM cases fulfilling the inclusion criteria were identified between 2012 and 2016. The highest number of cases occurred in the age group of 13 to 24 months. The median age was 2 years (interquartile range [IQR]: 1–5.75 years). The sex distribution was balanced, with 14 males and 16 females (Figure 1). Of these, 27 presented with cervical lymphadenitis, two of whom had additional pulmonary involvement. One patient had a SSTI, one had osteomyelitis, and one presented with isolated pulmonary NTM disease (Table 1). Immunodeficiency was not restricted to patients with other chronic diseases, explaining the higher proportion of immunodeficiency compared to chronic disease in the cohort.

**Table 1:** Distribution of clinical and epidemiological characteristics in pediatric patients with bacteriologically confirmed or clinically probable NTM disease.

		*N* (%)
BCG vaccinated		
	Yes	7 (23.3)
	No	22 (73.3%)
	Not available	1
Site of infection		
	Cervical lymphadenitis	25 (83.3)
	Cervical lymphadenitis with pulmonary involvement	2 (6.6)
	Osteomyelitis	1
	Pulmonary	1
	Skin	1
Histological evidence of granulomatous inflammation		
	Yes	26 (86.7)
	No	4 (13.3)
Exposure to animals		
	Yes	20 (66.6)
	No	10 (33.4)
Exposure to untreated water		
	Yes	11 (36.7)
	No	19 (63.3)
Underlying conditions		
	Any underlying chronic condition^a^	10 (33.4)
	Immunodeficiency (primary or secondary)^b^	12 (40.0)
	Primary immunodeficiency	1 (3.3)
	Secondary immunodeficiency or functional immune abnormality	11 (36.7)
	Other chronic disease without immunodeficiency^c^	4 (13.3)
	No underlying condition identified	16 (53.3)
Mantoux test		
	Positive	11 (36.7)
	Negative	19 (63.3)
Energy		
	Yes	9 (30.0)
	No	21 (70.0)

a Chronic disease was defined as a clinically diagnosed long-term comorbidity documented in the medical record.

b Immunodeficiency was analyzed as an independent variable and included both primary immunodeficiency disorders and secondary or functional immune abnormalities identified by immunological testing or clinical assessment.

c Other chronic disease without immunodeficiency included asthma bronchiale, periodic fever syndromes, polycythaemia, and thrombophlebitis.

**Figure 2:**
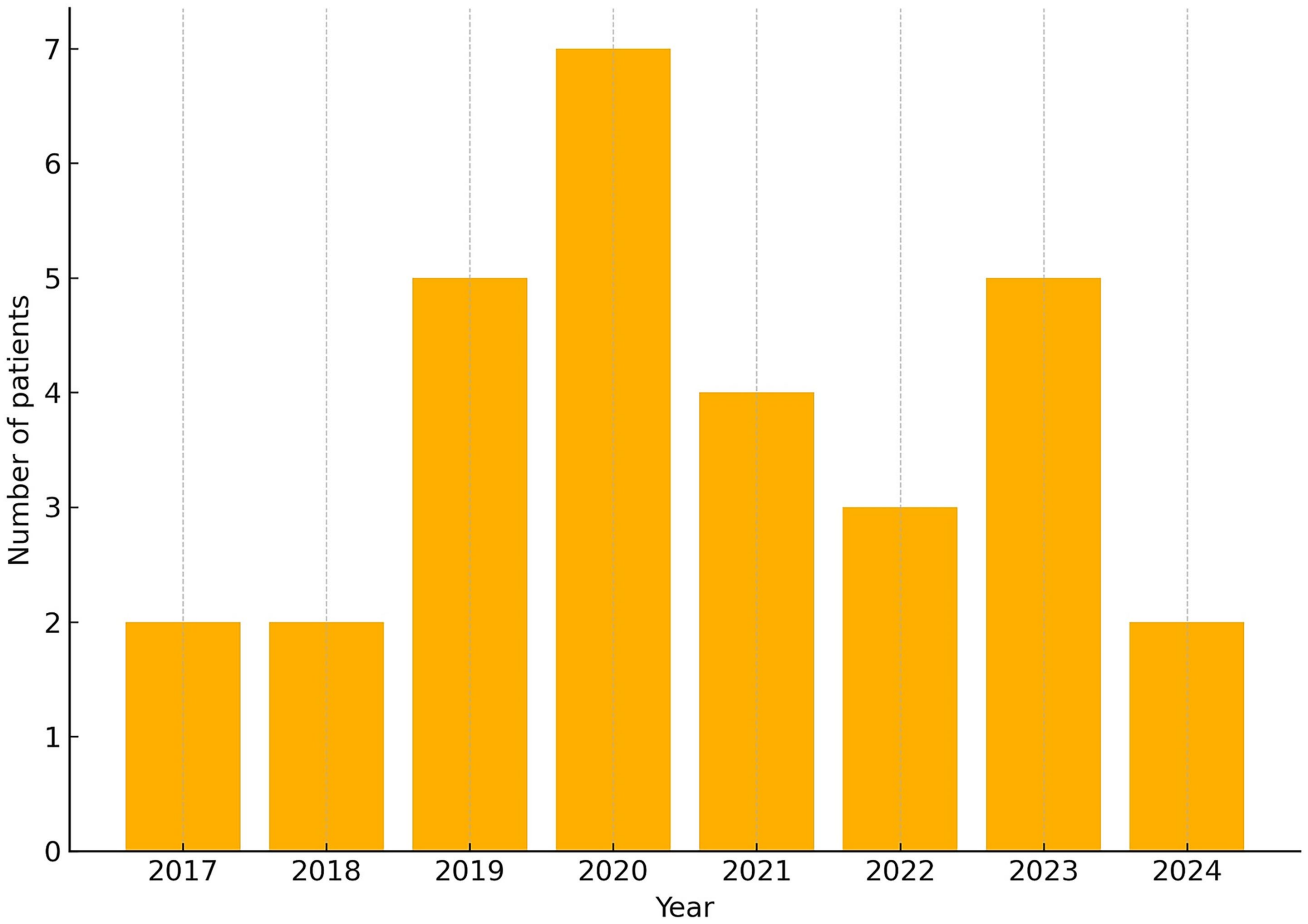
Annual number of pediatric NTM cases diagnosed at the National Institute for Pediatric Tuberculosis and Respiratory Diseases in Slovakia between 2017 and 2024. The figure represents absolute case counts rather than population-based incidence rates.

No confirmed pediatric NTM diseases fulfilling the inclusion criteria were identified during the period of mandatory BCG vaccination (until 31.12.2011). However, contrary to expectations of an earlier emergence of NTM cases following the cessation of BCG vaccination, the first patients were diagnosed in 2017 (Figure 2).

Nevertheless, 7 patients (23.3%) with confirmed NTM disease had received BCG vaccination. Among them, 4 had coexisting chronic conditions, including thrombophlebitis, polycythemia, periodic fever syndromes, asthma, and one case of Mendelian susceptibility to mycobacterial disease (MSMD), subtype–complete interferon-*γ* receptor 1 (IFN-γR1) deficiency (verified by homozygous mutation 523delT in exon 4 for IFN-γR1). These findings suggest that host factors, such as underlying immune or inflammatory disorders, may contribute to NTM disease even in the presence of BCG-induced protection.

In relation to potential environmental sources of infection, 20 patients reported exposure to animals and 11 patients reported exposure to untreated water. However, these potential sources were not further investigated to confirm the presence of NTM.

### 3.2 Diagnostics

Overall, NTM disease was bacteriologically confirmed at the patient level in 16 of 30 patients (53.3%). The remaining 14 patients fulfilled criteria for clinically probable NTM disease. A total of 79 clinical specimens were analyzed, obtained from all 30 enrolled patients (including both microbiologically confirmed and clinically probable NTM disease cases). Diagnostic yield varied by specimen type and laboratory method, as summarized in Table 2.

**Table 2:** Specimen-level diagnostic yield by specimen type and laboratory method.

Specimen type	No. of specimens analyzed	PCR positive *n* (%)	Culture positive *n* (%)	Smear positive *n* (%)
Lymph node biopsy	15	5 (33.3%)	5 (33.3%)	2 (13.3%)
Gastric aspirate	30	2 (6.7%)	1 (3.3%)	1 (3.3%)
Wound swab	6	3 (50.0%)	1 (16.7%)	1 (16.7%)
Laryngeal swab	25	0 (0%)	0 (0%)	0 (0%)
Sputum	1	0 (0%)	1 (100%)	1 (100%)
Bronchoalveolar lavage	1	1 (100%)	0 (0%)	0 (0%)
Bone marrow	1	0 (0%)	1 (100%)	0 (0%)
Total specimens	79	–	–	–

Percentages are calculated per specimen type. Individual patients could contribute more than one specimen; therefore, specimen-level positivity does not correspond directly to the number of microbiologically confirmed patients.

Histological evidence of granulomatous inflammation was observed in 26 out of 30 patients (86.7%).

In eight patients, the disease was caused by members of the *M. avium* complex, including *M. avium* in six cases and *M. intracellulare* in two cases. In seven additional patients, the presence of *Mycobacterium* species was confirmed without further species-level identification. In the patient with NTM-associated osteomyelitis, *M. arupense* was identified as the causative agent.

**Figure 3:**
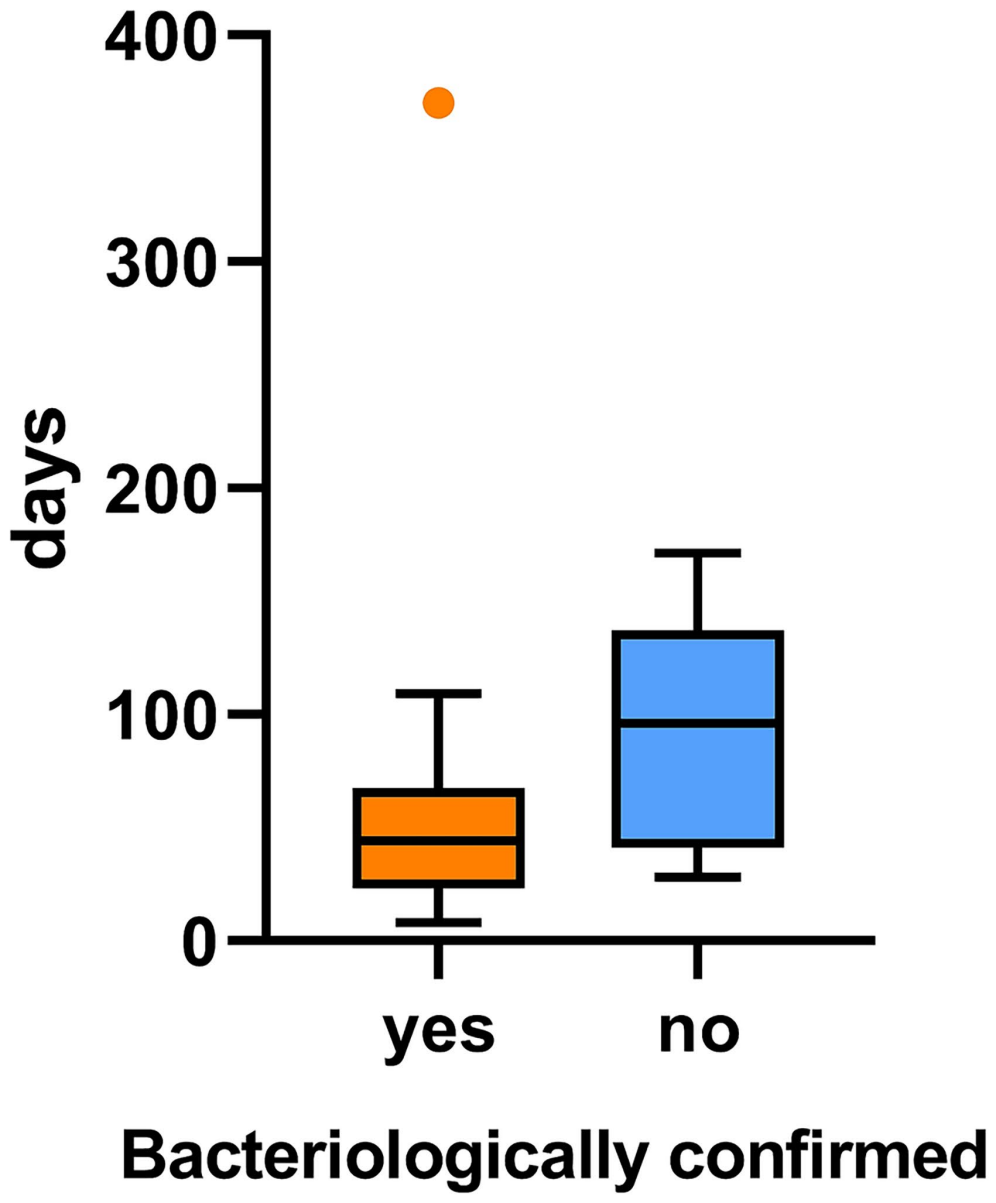
Difference in time to diagnosis in pediatric NTM disease by bacteriological confirmation status.

The median time from symptom onset to initiation of first NTM-directed intervention (surgical excision or antimycobacterial therapy) was 45 days (IQR: 31–109.5) across all patients (Figure 3). The median diagnostic delay was longer in bacteriologically negative cases compared with bacteriologically positive cases (100.0 vs. 40.5 days, median difference 59.5 days, 95% CI 23.5–116.5), although this difference did not reach statistical significance (*p* = 0.0648). However, the absence of statistical significance should not be interpreted as evidence of equivalence between groups.

### 3.3 Treatment

In the majority of patients with cervical lymphadenitis (26 out of 27) and in the patient with skin and SSTI (*n* = 1), surgical excision of the affected lymph nodes was performed. A total of 22 patients received antimicrobial therapy with a combination of rifampicin and clarithromycin after excision. The duration of treatment ranged from 1 to 20 months, with a median of 3.5 months (interquartile range [IQR]: 3–6 months). Four patients did not receive adjunct antimicrobial therapy. Treatment decisions were individualized and guided by the clinical course and presentation.

Three patients (one with cervical lymphadenitis and concurrent pulmonary involvement, one with osteomyelitis, and one with isolated pulmonary disease) were managed exclusively with antibiotic therapy. All three received a combination of rifampicin and clarithromycin, while the patient with osteomyelitis was additionally treated with ethambutol. The duration of treatment ranged from 4 to 6 months. Although ATS/IDSA guidelines for pulmonary NTM disease generally recommend treatment for at least 12 months after culture conversion, a shorter 6-month regimen was selected because of marked clinical improvement, mild disease course, and the very young age of the patients. A shorter duration was also intended to minimize long-term drug-related toxicity and unnecessary exposure to broad-spectrum antibiotics with consequent selective pressure for multidrug-resistant bacteria (2). No adverse effects related to antibiotic therapy were documented in any of the patients.

Follow-up data were available for 19 patients, with a follow-up duration ranging from 3 to 72 months. Fifteen patients had fully recovered without any persistent symptoms or complications following either surgical intervention or completion of antimicrobial treatment. Three patients with cervical lymphadenitis experienced recurrent upper respiratory tract infections. These episodes were considered potentially unrelated to NTM disease, as a direct causal association between prior NTM infection and recurrent upper respiratory tract infections has not been established and they likely reflect common pediatric morbidity. In one of these cases, this led to repeated adenoidectomy and tonsillectomy. Death was documented in one patient, who had been diagnosed with MSMD. The primary cause of death was acute miliary tuberculosis.

## 4 Discussion

This study provides a comprehensive overview of pediatric patients with NTM disease diagnosed at the National Institute for Pediatric Tuberculosis and Respiratory Diseases in Slovakia over a 13-year period. Our findings are consistent with previous reports indicating that cervical lymphadenitis caused by MAC in children aged 1 to 2 years represents the most common clinical manifestation of NTM disease in the pediatric population (7).

The observed temporal distribution of pediatric NTM cases should be interpreted with caution. The data presented reflect absolute numbers of diagnosed and referred cases rather than population-based incidence rates. Therefore, no firm conclusions regarding temporal trends or changes in incidence can be drawn. Several system-level factors may have contributed to the observed temporal pattern. Increased clinical awareness of pediatric NTM disease over time, particularly among pediatric infectious disease specialists and surgeons, likely led to improved recognition and referral of suspected cases. In parallel, the gradual implementation and wider availability of molecular diagnostic methods, including PCR-based assays, may have facilitated case confirmation in later years. Changes in referral patterns to the national tertiary center, with increasing centralization of complex pediatric mycobacterial cases, may also have influenced the number of diagnosed cases. Finally, evolving documentation and reporting practices over time could have contributed to improved case capture. Although similar temporal patterns following BCG discontinuation have been reported in other Central European countries, direct comparisons are limited by differences in surveillance systems, diagnostic criteria, referral structures, and the availability of population denominators (18, 19). An upward trend in case numbers since 2012 has been documented in a nationwide study of pulmonary mycobacterioses in Slovakia; however, the study did not include stratification by patient age, limiting its applicability to the pediatric population (20). Another important factor potentially contributing to the increasing number of pediatric NTM cases is the presence of underlying immune disorders (18). In our cohort, immunodeficiency was identified in 40% of patients. Slovakia ranks among the countries with the highest proportion of pediatric tuberculosis (TB) cases, with over 80% of affected children belonging to the Roma ethnic minority (21). Notably, in our cohort, no patients were of Roma origin. This striking contrast may, among other factors, support the theory of cross-immunity mechanisms among mycobacterial species, as proposed by previous study (22).

Patient-reported exposure to potential environmental risk factors revealed that 66.6% had contact with animals (including pets and farm animals), and 36.7% exposure to untreated water sources, as well as swimming pools. This information was collected as part of routine epidemiological anamnesis; however, determining whether these exposures represented true sources of infection would require targeted environmental microbiological sampling, which was not performed. Consequently, these variables serve solely as descriptive clinical characteristics rather than analytical predictors, and no statistical testing was conducted to evaluate their relationship with NTM disease. Prior studies have indicated that these factors are not statistically significant risk factors for NTM-related cervical lymphadenitis (8). In contrast, water exposure was considered the likely source of infection in one patient with a SSTI who developed a granulomatous lesion following a cut sustained while handling an aquarium. The presumed causative organism in this case is *M. fortuitum*, *M. chelonae* or *M. abscessus, the three primary SSTI-causing NTM*, for which the contaminated water is the most frequent source of infection (23).

An important methodological aspect of this study is the inclusion of patients without microbiological confirmation of NTM disease. This reflects the real-world diagnostic challenges in pediatric NTM diseases, where microbiological sensitivity is limited, particularly in extrapulmonary forms such as cervicofacial lymphadenitis. Importantly, stratification by microbiological confirmation demonstrated longer diagnostic delays in clinically probable cases, supporting the clinical relevance of this distinction. Pooling of confirmed and probable cases was applied only for descriptive epidemiological analyses and was justified by the shared clinical phenotype, management approach, and the small size of the cohort.

Despite being the most frequently collected specimen, gastric aspirates demonstrated very limited diagnostic yield, with positivity rates below 10% across all laboratory methods. This highlights the importance of obtaining tissue samples when feasible and involving multidisciplinary expertise to ensure accurate diagnosis. Notably, laryngeal swabs yielded no positive microbiological results, suggesting minimal diagnostic value in suspected pediatric NTM disease. The median time from symptom onset to diagnosis was 45 days, with a longer diagnostic delay observed in bacteriologically negative patients. Although this difference did not reach statistical significance, it reflects a consistent trend that is biologically plausible, as the absence of microbiological confirmation often complicates clinical decision-making and may delay initiation of targeted therapy. However, given the small sample size, the study lacked sufficient statistical power to detect modest differences. As a result, the non-significant *p* value should not be interpreted as evidence of equivalence between groups. Instead, the finding underscores the need for cautious interpretation and highlights the constraints of underpowered analyses in rare pediatric diseases. Also, these analyses were exploratory in nature and intended to generate hypotheses for future studies. The case of osteomyelitis caused by *M. arupense* illustrates the diagnostic challenges in extrapulmonary NTM disease. Despite initial surgical management and thorough testing, the diagnosis was confirmed only after a second operation and prolonged culture from bone marrow. This case underlines the need to consider NTM even in atypical presentations and the importance of repeated sampling when initial results are inconclusive (24).

In our cohort, 26 out of 27 patients with unilateral cervical lymphadenitis underwent surgical excision of the affected lymph nodes, consistent with current clinical practice that favours surgical intervention as the primary therapeutic approach. The majority of these patients also received adjunctive antimicrobial therapy with a combination of rifampicin and clarithromycin. The role of adjunctive antibiotic therapy in NTM lymphadenitis remains debated. Several studies have shown that surgical excision alone is associated with high cure rates and low recurrence when complete resection is achieved (4, 25). However, antibiotic therapy may be beneficial in cases with incomplete resection, extensive disease, or when surgery is contraindicated. Guidelines from the ATS/IDSA support both surgical and medical management, with the choice depending on individual clinical conditions (26).

This study has several limitations. First, the relatively small sample size and single-center setting may limit the generalizability of the findings to other populations. However, given that the majority of children with suspected NTM disease in Slovakia are referred to a single center, the low number of included cases reflects the overall rarity and low incidence of these diseases in the pediatric population rather than underreporting or selection bias. Second, strain-level identification and genotyping of NTM species were not performed for several patients, which precludes more detailed epidemiological inferences. Although our dataset included several clinical phenotypes, meaningful statistical stratification was not feasible due to the markedly uneven distribution of cases. Cervical lymphadenitis accounted for the vast majority of diagnoses (25/30 patients), whereas other phenotypes (pulmonary disease, osteomyelitis, and SSTI) were represented by only one or two cases each. Any comparative statistical analysis across such disproportionately small subgroups would be methodologically inappropriate and prone to highly unstable or misleading estimates. For this reason, stratified analyses were limited to descriptive reporting only. To enable robust assessments of clinical heterogeneity, future studies will require substantially larger cohorts with a more balanced representation of phenotypes. In low-incidence countries such as Slovakia, this can realistically be achieved only through multicenter or international collaborative datasets, which would provide sufficient statistical power and improve the generalizability of phenotype-specific comparisons. In addition, information on animal and water exposures was based on caregiver recall and may therefore be subject to recall bias. Follow-up data were incomplete, which may have limited the detection of late or mild persistent symptoms and led to underascertainment of long-term complications. Regarding diagnostic delay, time to first tissue sampling would represent a clinically informative complementary metric, these data were not uniformly available in this retrospective cohort and could therefore not be reliably analyzed.

## Data Availability

The raw data supporting the conclusions of this article will be made available by the authors, without undue reservation.
